# Sodium-Hydrogen Exchanger Isoform-1 Inhibition: A Promising Pharmacological Intervention for Resuscitation from Cardiac Arrest

**DOI:** 10.3390/molecules24091765

**Published:** 2019-05-07

**Authors:** Raúl J. Gazmuri, Jeejabai Radhakrishnan, Iyad M. Ayoub

**Affiliations:** 1 Resuscitation Institute, Rosalind Franklin University of Medicine and Science, Section of Critical Care Medicine, Captain James A. Lovell Federal Health Care Center, North Chicago, IL 60064, USA; 2 Resuscitation Institute, Rosalind Franklin University of Medicine and Science, North Chicago, IL 60064, USA; jeejabai.radhakrishnan@rosalindfranklin.edu (J.R.); iyad.ayoub@rosalindfranklin.edu (I.M.A.)

**Keywords:** cardiopulmonary resuscitation, energy metabolism, ischemia, mitochondria, myocardium, reperfusion injury, sodium calcium exchanger, sudden cardiac arrest, ventricular function

## Abstract

Out-of-hospital sudden cardiac arrest is a major public health problem with an overall survival of less than 5%. Upon cardiac arrest, cessation of coronary blood flow rapidly leads to intense myocardial ischemia and activation of the sarcolemmal Na^+^-H^+^ exchanger isoform-1 (NHE-1). NHE-1 activation drives Na^+^ into cardiomyocytes in exchange for H^+^ with its exchange rate intensified upon reperfusion during the resuscitation effort. Na^+^ accumulates in the cytosol driving Ca^2+^ entry through the Na^+^-Ca^2+^ exchanger, eventually causing cytosolic and mitochondrial Ca^2+^ overload and worsening myocardial injury by compromising mitochondrial bioenergetic function. We have reported clinically relevant myocardial effects elicited by NHE-1 inhibitors given during resuscitation in animal models of ventricular fibrillation (VF). These effects include: (a) preservation of left ventricular distensibility enabling hemodynamically more effective chest compressions, (b) return of cardiac activity with greater electrical stability reducing post-resuscitation episodes of VF, (c) less post-resuscitation myocardial dysfunction, and (d) attenuation of adverse myocardial effects of epinephrine; all contributing to improved survival in animal models. Mechanistically, NHE-1 inhibition reduces adverse effects stemming from Na^+^–driven cytosolic and mitochondrial Ca^2+^ overload. We believe the preclinical work herein discussed provides a persuasive rationale for examining the potential role of NHE-1 inhibitors for cardiac resuscitation in humans.

## 1. Introduction

Out-of-hospital sudden cardiac arrest is a major public health problem worldwide with close to 360,000 cases assessed every year by Emergency Medical Services (EMS) in the United States. In approximately half of these cases, cardiopulmonary resuscitation (CPR) is attempted, and of these only 9% survive the episode and return to their pre-arrest condition with adequate neurological function [1]. Efforts to improve survival emphasize early recognition of cardiac arrest, activation of the EMS system, delivery of high-quality CPR, and optimal post-resuscitation care. The disappointing outcome occurs despite a highly organized and widespread effort to deliver these interventions promptly and efficiently.

The crucial first step in the resuscitation effort is the restoration of cardiac activity, which depends largely on the ability of CPR to generate sufficient coronary blood flow to ameliorate myocardial ischemia and establish the metabolic conditions required for the return of an electrically organized and mechanically effective cardiac activity. However, conventional CPR is unable to generate more than 10% to 20% of the coronary blood flow [2,3,4], constraining the resuscitation effort to proceed without reversing but only mitigating the severity of myocardial ischemia [2,4,5,6]. Moreover, this minimal blood flow reintroduces oxygenated blood to an ischemic myocardium triggering what is known as reperfusion injury, further accentuating the severity of ischemic injury. Mitochondria are at the center of reperfusion injury, with their bioenergetic function affected primarily by the generation of reactive oxygen species and by Ca^2+^ overload followed by eventual opening of the mitochondrial permeability transition pore and collapse of the proton-motive force required for ATP synthesis [7,8,9,10].

We have conducted preclinical research on various animal models of cardiac arrest focusing for the past two decades on the hypothesis that resuscitation outcomes can be improved by interventions able to attenuate myocardial reperfusion injury. We have examined various strategies [5,6,10,11,12,13,14,15,16,17,18,19,20,21,22,23,24,25,26,27] and so far identified erythropoietin [18,23,24] and inhibitors of the sodium-hydrogen exchanger isoform 1 (NHE-1) [5,6,11,12,13,14,15,16,17,19,20,21,22,27] as promising interventions.

The present review is focused on our work exploring the myocardial effects of NHE-1 inhibitors during cardiac resuscitation discussing mechanisms and physiological effects that we propose are relevant for clinical translation.

## 2. Rationale for Targeting NHE-1 during Cardiac Resuscitation

**Figure 1:**
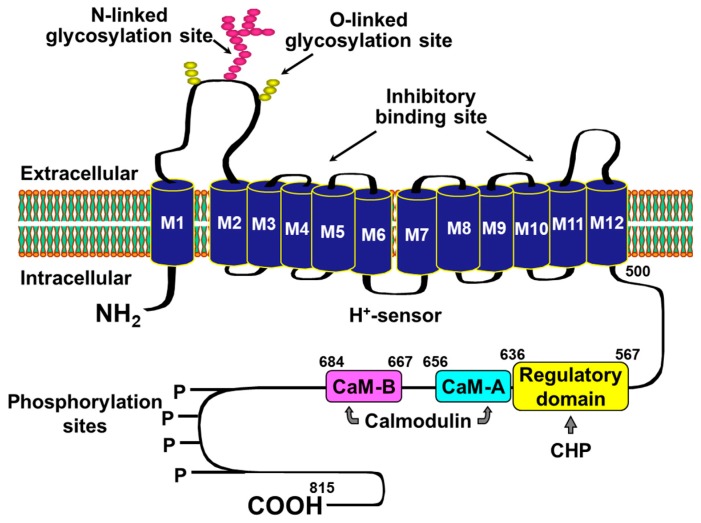
Putative topologic model of an 815-amino acid Na^+^-H^+^ exchanger isoform-1. Depicted are 12 transmembrane-spanning segments and a 315-amino acid cytoplasmic hydrophilic carboxyl terminus domain (COOH). The transmembrane domain includes the intracellular H^+^-sensor site, the functional ion exchange site, and the site where inhibitors bind. The cytoplasmic domain includes sites for regulation via phosphorylation-dependent (P) and phosphorylation-independent mechanisms. Depicted are CaM-A and CaM-B representing high- and low-affinity binding sites for the Ca^2+^ binding protein calmodulin and CHP, denoting calcineurin homologous protein. Not shown are additional regulatory sites in the cytoplasmic domain for the extracellular signal regulated kinase (ERK1/2), p38 mitogen-activated protein kinase (MAPK), ribosomal S6 kinase (RSK), RhoA kinase, and the tyrosine kinase 2 (Pyk2).

**Figure 2:**
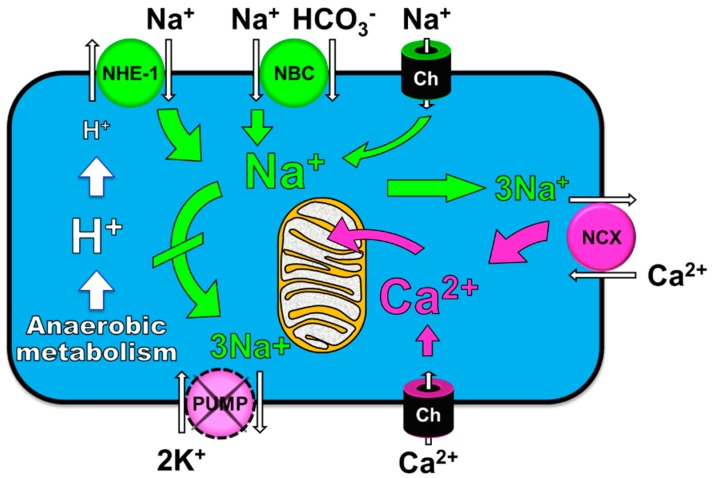
Rendition of a cardiomyocyte during ischemia and reperfusion depicting Na^+^-induced cytosolic and mitochondrial Ca^2+^ overload consequent to ischemia generating intracellular acidosis while the Na^+^-K^+^ pump is inhibited. Ch, channel; NBC, Na^+^-HCO_3_^−^ co-transporter; NCX, Na^+^-Ca^2+^ exchanger; NHE-1, sodium– hydrogen exchanger isoform-1.

NHE-1 is a member of a large family of NHE exchangers [28]. NHE-1 is the so-called housekeeping isoform and is ubiquitously expressed in plasma membrane of most tissues [29,30,31]. NHE-1 is the primary, if not the only, isoform expressed in mammalian cardiomyocytes [32]. It has 815 amino acids with ~500 forming a 12 transmembrane spanning domain responsible for the Na^+^-H^+^ exchange and the remaining 315 amino acids forming a cytoplasmic domain containing various regulatory sites (Figure 1). NHE-1 is activated by intracellular acidosis promoting an allosterically regulated electroneutral Na^+^-H^+^ exchange that enables exit of intracellular H^+^ in exchange for extracellular Na^+^ [31,33]. When cardiac arrest occurs, cessation of blood flow prompts tissue ischemia with rapid development of intracellular acidosis, which is particularly intense in the heart given its high metabolic rate [34,35,36]. Thus, the exit of H^+^ is accompanied by the entry of Na^+^ to cardiomyocytes. During the ensuing resuscitation effort, the reperfusion that accompanies the resuscitation effort washes-out H^+^ accumulated in the extracellular space (i.e., during the preceding no-flow interval of cardiac arrest) and intensifies the Na^+^-H^+^ exchange along with the corresponding cytosolic Na^+^ entry [12,27,31,37]. Yet, the extrusion of Na^+^ from the cytosol during ischemia is limited consequent to inhibition of the Na^+^-K^+^ pump [38]. As result, there is progressive and prominent Na^+^ accumulation in the cytosol [31,39,40]. Additional Na^+^ may enter the cytosol through Na^+^ channels and the Na^+^-HCO_3_^−^ co-transporter (Figure 2). 

The cytosolic Na^+^ overload, in turn, decreases cytosolic Ca^2+^ exit through the Na^+^-Ca^2+^ exchanger and eventually causes cytosolic Ca^2+^ influx through reverse mode operation of the exchanger leading to cytosolic Ca^2+^ overload [41] and subsequent mitochondrial Ca^2+^ entry (Figure 2); a process which involves the mitochondrial Ca^2+^ uniporter with the mitochondrial Na^+^-Ca^2+^ exchanger involved in Ca^2+^ efflux [42]. Mitochondria can buffer large amounts of Ca^2+^ in its matrix. Yet, when this buffering capacity is overwhelmed, free mitochondrial Ca^2+^ rises eventually saturating the mitochondrial Na^+^-Ca^2+^ exchanger leading to detrimental mitochondrial Ca^2+^ overload [42]. Excess mitochondrial Ca^2+^ worsens cell injury in part by compromising oxidative phosphorylation, releasing pro-apoptotic factors, and by lowering the threshold for opening of the mitochondrial permeability transition pore [10,20,43].

## 3. Functional Effects of NHE-1 Inhibition during Cardiac Resuscitation

Our laboratory [5,6,11,12,13,14,15,16,17,19,20,21,22,27], and a few others [44,45,46,47,48], have provided robust preclinical support for a potential beneficial effects of NHE-1 inhibition during cardiac resuscitation, showing myocardial effects that—if translated to humans during cardiac resuscitation—could markedly increase the rate of successful resuscitation and subsequent survival with adequate neurological function.

### 3.1. Effects on Left Ventricular Distensibility during VF-Induced Cardiac Arrest

**Figure 3:**
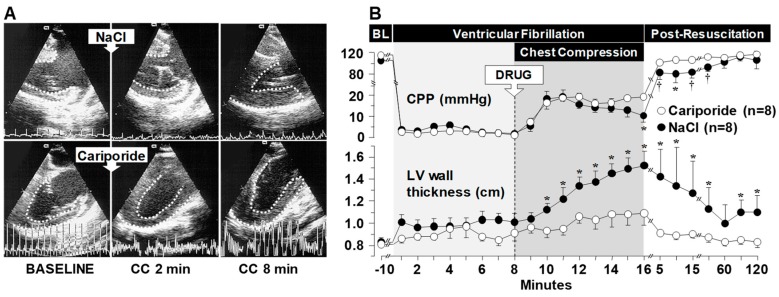
(**A**) Left ventricular wall thickening during chest compression (CC) in an NaCl-treated pig (upper frames) but not in a cariporide-treated pig (lower frames), measured by transesophageal echocardiography at the end of diastole at baseline and between chest compressions (CC) at 2 and 8 min of CPR. (**B**) Progressive decreases in the coronary perfusion pressure (CPP) coincident with progressive left ventricular (LV) wall thickening in NaCl-treated pigs but not in cariporide-treated pigs. NaCl or cariporide (drug, 3 mg/kg) was given immediately before starting chest compression. Mean ± SEM. * *p* < 0.05, ^†^ *p* < 0.001 *vs*. cariporide by one-way ANOVA (Reproduced with permission from Ayoub et al. [14]).

Left ventricular distensibility is important for adequate preload. During cardiac resuscitation, left ventricular distensibility gradually declines compromising the ability of cardiac compression to generate forward blood flow [49]. Our NHE-1 work started in an isolated rat heart model of ventricular fibrillation (VF) that we had developed to simulate sudden cardiac arrest and resuscitation [50]. The NHE-1 inhibitor cariporide was infused to yield a 10 mmol/L concentration in the coronary circuit during the interval of simulated cardiac resuscitation [11]. Cariporide markedly attenuated left ventricular pressure increases during the interval of simulated resuscitation, indicative of NHE-1 inhibition preventing reductions in left ventricular distensibility. Following defibrillation and restoration of cardiac activity, the beneficial effect of cariporide on left ventricular distensibility persisted preventing a leftward shift of the end-diastolic pressure-volume curve. These observations prompted work in an intact rat model of VF and close-chest resuscitation [11] and subsequently in a translationally more relevant swine model of VF and closed-chest CPR [14]. In the swine experiments, cariporide given in bolus dose of 3 mg/kg immediately before starting chest compression had an impressive effect on left ventricular distensibility during CPR evidenced by preservation of left ventricular cavity size and wall thickness (Figure 3A,B). The preservation of left ventricular distensibility had a hemodynamically favorable effect during chest compression preventing the decline of coronary perfusion pressure as it occurred in control animals (Figure 3B). Coronary perfusion pressure drives coronary blood flow and therefore the effect of NHE-1 inhibition preserving left ventricular distensibility and the coronary perfusion pressure resulted in a higher resuscitability rate compared to controls (8/8 vs. 2/8; *p* < 0.05) [14].

The preceding observations supported the concept that a more distensible left ventricle would allow a larger volume of blood to fill the left ventricular cavity before each chest compression resulting in a large volume of blood to be ejected during compression. This effect would be expected to enhance the hemodynamic efficacy of chest compression and—in turn—explain the increased coronary perfusion pressure and higher resuscitability observed in the swine model [14]. To further explore this potential mechanism, we conducted studies in an intact rat model of VF and CPR. We measured cardiac output along with regional organ blood flows using fluorescent microspheres during chest compression while varying the depth of chest compression during resuscitation from VF [5].

We conducted two series of experiments in which rats were subjected to 10 min of untreated VF followed by 8 min of chest compression before attempting defibrillation with the compression depth adjusted to maintain an aortic diastolic pressure between 26 and 28 mmHg in the first series and between 36 and 38 mmHg in the second series. Within each series, rats were randomized to receive a bolus of cariporide (3 mg/kg) or NaCl 0.9% (vehicle-control) before starting chest compression.

**Figure 4:**
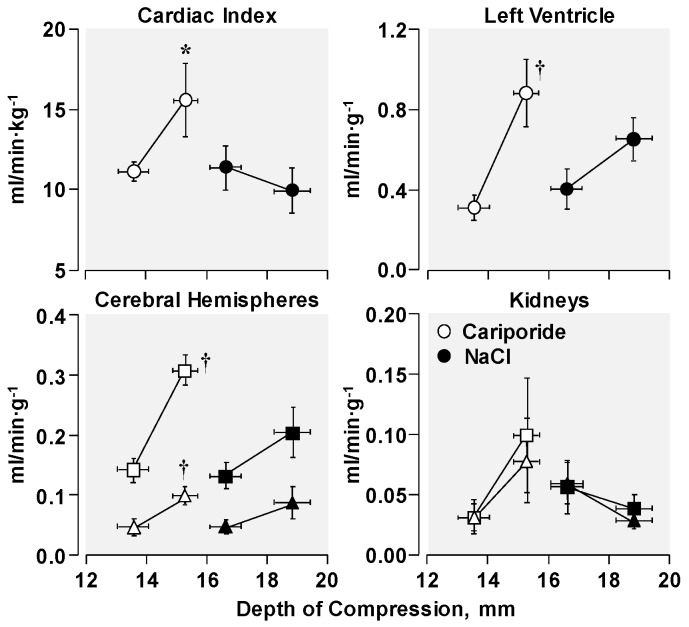
Leftward shift of the flow-depth relationship during chest compression elicited by cariporide in rats during VF. Rats were randomized to receive a bolus of cariporide or NaCl before starting chest compression. The first symbol represents data from series 1 and the second symbol from series 2. For paired organs, triangles denote right and squares left. Values are mean ± SEM; * *p* < 0.05 vs. NaCl by one-way ANOVA in series 2; ^†^ *p* < 0.01 vs. series 1 within each treatment group by one-way ANOVA (Adapted with permission from Kolarova et al. [5]).

In rats that received cariporide, higher cardiac output and higher organ blood flows (including heart and brain) were observed for a given compression depth (Figure 4). Thus, as hypothesized, cariporide shifted the relationship between blood flow and compression depth to the left as a result of maintaining left ventricular distensibility, confirming a novel concept in resuscitation whereby a pharmacological intervention could increase the hemodynamic efficacy of chest compression.

**Figure 5:**
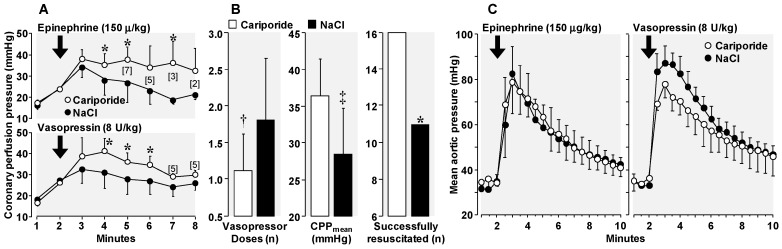
(**A**) Coronary perfusion pressure during chest compression in rats that received epinephrine (upper panel) or vasopressin (lower panel). Within each series, 16 rats were randomized to receive immediately before starting chest compression a 3 mg/kg bolus of cariporide or 0.9 % NaCl. With cariporide several rats experienced spontaneous defibrillation. The numbers in brackets indicate rat that remained in ventricular fibrillation. * *p* < 0.05 vs. NaCl by one-way ANOVA. (**B**) Combined data from both the epinephrine and vasopressin series in rats randomized to cariporide (*n* = 16) or NaCl (*n* = 16). CPPmean = Coronary perfusion pressure averaged through min 3 and 8 of chest compression. * *p* < 0.05 vs. NaCl by Fisher’s exact test; ^†^ *p* < 0.005, ^‡^ *p* < 0.0005 vs. NaCl by one-way ANOVA. (**C**) Aortic pressure during *in situ* perfusion. The vasopressor dose was given as a bolus at two minutes of low-flow perfusion. Either cariporide or 0.9% NaCl was infused throughout the low-flow perfusion state in both epinephrine (*n* = 8) and vasopressin (*n* = 8) series (adapted with permission from Kolarova et al. [17]).

Leveraging on this blood flow effect, we anticipated a positive interaction between NHE-1 inhibitors and vasopressor agents; i.e., for a higher blood flow generated by chest compressions in the presence of an NHE-1 inhibitor, the same increase in peripheral vascular resistance elicited by a particular vasopressor agent would be expected to generate a higher blood pressure and a higher coronary perfusion pressure aiding successful resuscitation. This effect, however, presumes that NHE-1 inhibitors lack or have minimal vasodilatory effects. We examined this interaction in our rat model of VF and chest compression [17]. We conducted two series of 16 experiments each, using epinephrine in one series and vasopressin in the other. Within each series, rats were randomized to receive a bolus of cariporide (3 mg/kg) or NaCl 0.9% (vehicle-control) immediately before starting chest compression with the vasopressor agents (epinephrine, 150 µg/kg or vasopressin, 8 U/kg) given as bolus doses at minimum intervals of 2 min to maintain the aortic diastolic pressure above 25 mmHg during chest compression. A significantly higher coronary perfusion pressure was generated when either vasopressor agent was given in rats that had received cariporide (Figure 5A). This favorable effect of cariporide reduced the number of vasopressor doses required and promoted higher resuscitation rates (Figure 5B).

We then examined whether cariporide had a direct vascular effect. For this purpose, in a similar rat model, we cannulated and perfused *in situ* the descending aorta with a Krebs-Henseleit solution at a flow rate titrated to generate an aortic pressure between 30 and 35 mmHg. This flow corresponded to ~20 mL/min. In addition, a 0.9% NaCl solution containing cariporide was infused and compared to NaCl control. Bolus administration of either epinephrine or vasopressin elicited—as expected—a transient aortic pressure increase of magnitude close to 80 mmHg. The effect was the same in the presence or absence of cariporide when vasopressin was administered but of smaller but not statistically significant magnitude when epinephrine was administered (Figure 5C). Thus, these studies did not support a direct vascular effect of cariporide.

These effects on coronary perfusion pressure are important. If translated clinically, they could be highly impactful because only small increases in coronary perfusion pressure are required to have a significant effect on resuscitability [3].

### 3.2. Effects on Ventricular Fibrillation, Defibrillation, and Post-Resuscitation Electrical Stability

In our intact rat model of VF and chest compression, spontaneous defibrillation frequently occurs after 7 to 9 min of chest compression in animals treated with cariporide but not in control animals [11]. This phenomenon was also reported by Wann et al. in a similar rat model of VF [51]. However, this effect has not been reported in larger animal models. Small size hearts—as in rats—handle VF differently. When electrically induced, prolonged electrical stimulation is required (e.g., ~3 min in rats) before self-sustained VF can be induced; otherwise VF reverts spontaneously to an organized rhythm. Small hearts enable a fibrillatory front to travel a much shorter distance such that its leading edge typically finds its trailing edge in refractory period precluding the reentry of the fibrillatory front required to maintain VF. Yet, after a period of myocardial ischemia; e.g., 3 min, conduction velocity slows down allowing the trailing edge to come off it refractory period enabling the arriving leading edge of the fibrillatory front to depolarize the myocardium leading to self-sustained VF [52]. Spontaneous defibrillation in our rat model is typically preceded by increases in the amplitude and frequency of the VF waveform [11,15,51]; an effect that is associated with improved myocardial energy state [36]. Thus, spontaneous defibrillation in the presence of cariporide in our rat model most likely reflects a beneficial effect of cariporide on myocardial energy metabolism; an effect we subsequently demonstrated in a larger animal model and is discussed later [6]. Another prominent effect observed in the presence of cariporide is suppression of ventricular ectopic activity and episodes of refibrillation post-resuscitation; an effect that typically occurs clinically during the early post-resuscitation period and is responsible for re-arrest [11,14,16,21]. In a swine model, this effect was associated with preservation of the action potential duration [14]; an effect that would reduce the risk of reentry [21]. Clinical translation of this effect would be highly impactful in preventing re-arrests in out-of-hospital cardiac arrest victims who are initially resuscitated and are in route to a hospital.

### 3.3. Effects on Post-Resuscitation Myocardial Function

**Figure 6:**
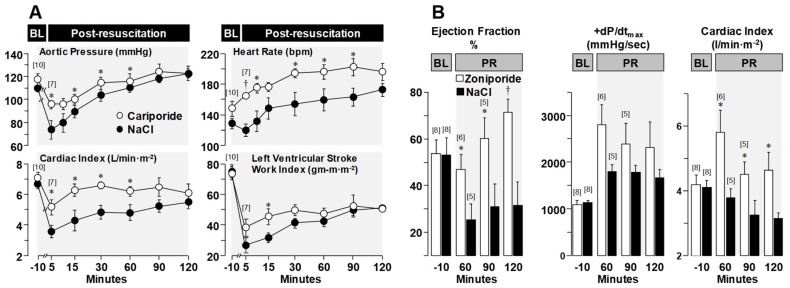
Baseline (BL) and post-resuscitation (PR) left ventricular and hemodynamic function. Numbers in brackets indicate sample size. Values are mean ± SEM. (**A**) Closed-chest swine model of VF and conventional cardiopulmonary resuscitation in which 20 pigs were randomized to receive cariporide or NaCl control during resuscitation from VF. * *p* < 0.05; ^†^ *p* < 0.001 vs. control by one-way ANOVA (adapted with permission from Ayoub et al. [21]). (**B**) Open-chest swine model of VF and resuscitation by extracorporeal circulation in which 16 pigs were randomized to receive zoniporide or NaCl control during resuscitation from VF. * *p* < 0.05, ^†^ *p* < 0.01 vs. NaCl. In both series administration of the NHE-1 inhibitor during cardiopulmonary resuscitation had a marked beneficial effect on post-resuscitation myocardial function (adapted with permission from Ayoub et al. [6]).

**Figure 7:**
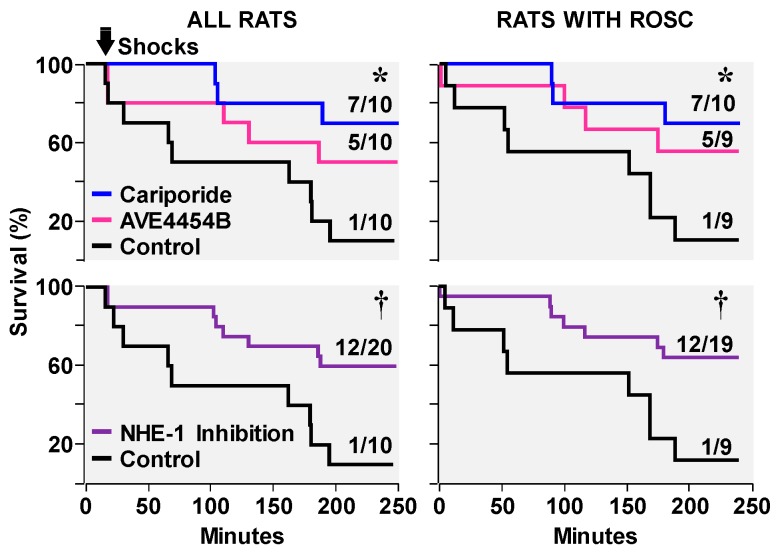
Survival curves in rats that received cariporide, AVE4454B, or control solution at the start of chest compression during resuscitation from VF. Shown are survival curves for all rats and survival curves only for rats that had return of spontaneous circulation (ROSC) after defibrillation (Shocks). Top graphs depict survival for the individual interventions. Bottom graphs depict survival for the cariporide and AVE4454B groups combined (NHE-1 Inhibition). ∗ *p* < 0.01 vs. control by Gehan-Breslow analysis using Holm-Sidak’s test for multiple comparisons; ^†^ *p* = 0.01 vs. control by Gehan-Breslow analysis (adapted with permission from Radhakrishnan et al. [22]).

Despite full restoration of coronary blood flow after the return of spontaneous circulation, variable degrees of left ventricular systolic dysfunction commonly occur. This phenomenon—known as myocardial stunning—is reversible. However, reversibility may take hours or days and contingent on its severity may compromise hemodynamic function and survival [53,54,55]. Diastolic dysfunction also occurs in the post-resuscitation period [11,14] and is linked to the same pathophysiological abnormalities responsible for decreased distensibility; namely, diastolic Ca^2+^ overload and energy deficit precluding full relaxation of cardiomyocytes. Administration of NHE-1 inhibitors during CPR in our animal models consistently lessens post-resuscitation left ventricular systolic and diastolic dysfunction. Figure 6 shows lesser left ventricular dysfunction with better hemodynamic function in 20 pigs randomized to receive a bolus of cariporide (3 mg/kg) or vehicle control after 6 min of untreated VF coincident with the start of chest compression [21], and in another study of 16 pigs randomized to receive a bolus of zoniporide (3 mg/kg) or vehicle control after 8 min of untreated VF coincident with the start of resuscitation using extracorporeal circulation [6]. Improved post-resuscitation myocardial function associated with NHE-1 inhibition leads to improved survival during the early post-resuscitation interval [13,22]. However, not all NHE-1 inhibitors seem to have the same effect. In a rat model of VF and conventional CPR, cariporide was more effective in improving short term survival than the newer compound AVE4454B [22] (Figure 7).

The beneficial myocardial effects of NHE-1 inhibitors have also been observed when given after return of spontaneous circulation. Lin et al. used a piglet model of asphyxial cardiac arrest and administered the NHE-1 inhibitor sabiporide 15 min after return of spontaneous circulation reporting a beneficial effect on left ventricular ejection fraction and hemodynamic function [48].

### 3.4. Amelioration of Adverse Epinephrine Effects

The rationale for routine epinephrine administration during cardiac resuscitation is to increase the coronary perfusion pressure and thereby coronary blood flow by promoting peripheral vasoconstriction given the limited capability of chest compression to generate adequate systemic blood flow. Thus, epinephrine increases the aortic blood pressure, which is the main driver of coronary perfusion. The vasoconstrictive effect of epinephrine is mediated through α_1_- and α_2_-adrenoceptors. However, epinephrine also activates β1- and β2-adrenoceptors. These α_1_-, β1-, and β2-adrenoceptors increase myocardial oxygen consumption; an undesirable effect during cardiac resuscitation when the heart is ischemic [2,56,57]. In a recent large randomized clinical trial, epinephrine increased by fourfold the rate of initial resuscitation; however, greater post-resuscitation deaths and worse neurologic outcome precluded the early beneficial effect of epinephrine to be sustained over time [58]. Our work suggests that some of the adverse effects of epinephrine may be neutralized by NHE-1 inhibition. In a swine model of VF and closed-chest resuscitation, the use of cariporide in a resuscitation protocol that included use of epinephrine resulted in higher resuscitation rates, fewer episodes of VF post-resuscitation, and lesser post-resuscitation myocardial dysfunction [16].

## 4. Cellular Mechanisms of the Observed Functional Benefits

As shown in Figure 2, activation of NHE-1 during ischemia and reperfusion have two immediate effects; i.e., H^+^ exit attenuating intracellular acidosis and Na^+^ entry leading to cytosolic and mitochondrial Ca^2+^ overload given the inability of the Na^+^-K^+^ pump to extrude Na^+^ from the cytosol. As also shown in Figure 2, other ports of entry exist for Na^+^, including Na^+^ channels—expected to open during VF (consequent to action potential activation). 

The cytosolic and mitochondrial Ca^2+^ overload adversely affects myocardial function by compromising mitochondrial bioenergetic function (i.e., ability to regenerate ATP in the presence of oxygen) and by precluding relaxation of cardiomyocytes. This last effect results from the alluded cytosolic Ca^2+^ overload and also from the inability of the energy-requiring sarcoplasmic reticulum Ca^2+^-ATPase to pump Ca^2+^ from the cytosol into the sarcoplasmic reticulum as part of the normal cardiomyocyte Ca^2+^ cycling. Altogether, these effects explain the left ventricular wall thickening with reduction in cavity size (i.e., decreased distensibility) during VF and post resuscitation left ventricular systolic and diastolic dysfunction. In a series of experiments, discussed below, we documented the actions of NHE-1 inhibitors attenuating these abnormalities and the corresponding functional effects. We first used our rat model of VF and closed-chest resuscitation to examine the effects of NHE-1 inhibition and Na^+^ channel blockade—interventions collectively referred to as “Na^+^-limiting interventions”—on intracellular Na^+^ content, mitochondrial Ca^2+^ content, cardiac function, and plasma levels of cardio-specific troponin I (cTnI) [19]. Measurements were made in hearts harvested at baseline, at 15 min of untreated VF, at 15 min of VF with chest compressions provided during the last 5 min of VF, and at 60 min post-resuscitation. Rats from the latter two time-events were randomized to receive a Na^+^-limiting intervention immediately before starting chest compression or vehicle control. The Na^+^-limiting interventions included the NHE-1 inhibitor AVE4454B (1 mg/kg), lidocaine (5 mg/kg), and the combination of AVE4454B and lidocaine.

**Figure 8:**
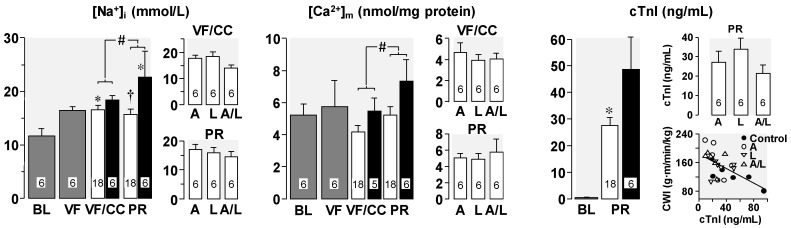
Left ventricular intracellular Na^+^ ([Na^+^]_i_), mitochondrial Ca^2+^ ([Ca^2+^]_m_), and cTnI (**C**) at baseline (BL), at 15 min of untreated VF, at 15 min of VF with chest compression during the last 5 min (VF/CC), and at 60 min post-resuscitation (PR). Black bars denote control rats and white bars denote rats treated with Na^+^-limiting interventions. Also shown are the effects of individual Na^+^-limiting interventions. A, AVE4454B and lidocaine vehicle; L, lidocaine and AVE4454B vehicle; and A/L, AVE4454 and lidocaine. Mean ± SEM. ∗ *p* < 0.05 vs. BL by Kruskal-Wallis one-way ANOVA on ranks using Dunn’s Method for multiple comparisons; ^†^ *p* < 0.05 vs. control by Student’s t-test in PR groups; ^#^two-way ANOVA using time factor (VF/CC *vs*. PR) and treatment factor (control vs. Na^+^-limiting interventions) was significant for treatment factor (*p* = 0.013) for [Na^+^]_i_ and for both, time factor (*p* = 0.045) and treatment factor (*p* = 0.021) for ([Ca^2+^]_m_. Also shown is an attenuation of post-resuscitation cTnI increased with the Na^+^-limiting interventions (∗ *p* < 0.05 vs. control by Student’s t-test) and an inverse relationship between cardiac work index (CWI) and cTnI levels (*r* = 0.58, *n* = 24, *p* < 0.01) (adapted with permission from Wang et al. [19]). Please confirm if it is necessary to explain # in the figure.

As shown in Figure 8, limiting sarcolemmal Na^+^ entry attenuated increases in cytosolic Na^+^ and mitochondrial Ca^2+^ overload during chest compression and the post-resuscitation period. This effect was associated with attenuation of post-resuscitation cTnI increase with the level inversely proportional to cardiac work. In a previous study using an isolated perfused rat heart model we reported that VF contributed independently to left ventricular Na^+^ overload and speculated that the fibrillatory activity—by opening of Na^+^ channels—could have been the contributing mechanism [12].

We also used an open-chest pig model of electrically-induced VF and extracorporeal circulation to study the effects of inhibiting NHE-1 on myocardial energy metabolism under conditions of controlled coronary perfusion pressure [6]. For this study, VF was induced by epicardial delivery of an alternating current and left untreated for 8 min. After this interval, extracorporeal circulation was started and the extracorporeal blood flow adjusted to maintain a coronary perfusion pressure at 10 mmHg for 10 min before attempting defibrillation. The target coronary perfusion pressure was chosen to mimic the low coronary perfusion pressure generated by closed-chest resuscitation. Two groups of eight pigs each were randomized to receive the NHE-1 inhibitor zoniporide (3 mg/kg) or vehicle control as a right atrial bolus immediately before starting extracorporeal circulation. Similar to previous studies using the NHE-1 inhibitor cariporide [14], zoniporide also prevented reductions in left ventricular distensibility during the interval of VF and extracorporeal circulation, which in control pigs was characterized by progressive reductions in cavity size and progressive thickening of the left ventricular wall. Importantly, these effects occurred without changes in coronary blood flow or coronary vascular resistance indicating that the favorable myocardial effects of NHE-1 inhibition during resuscitation are not likely to be mediated through increases in blood flow and oxygen availability.

**Figure 9:**
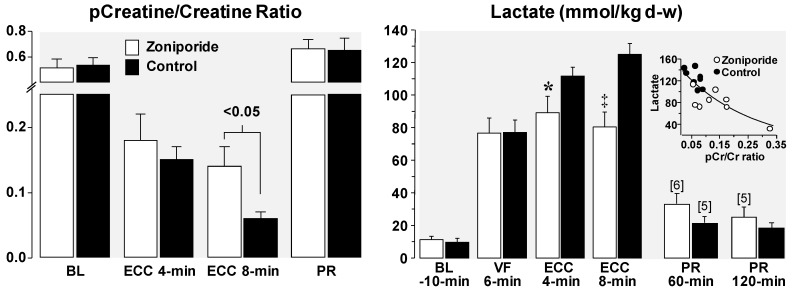
Myocardial measurements in pigs randomly assigned to receive 3 mg/kg of zoniporide or 0.9% NaCl control after 8 min of untreated ventricular fibrillation (VF) before starting extracorporeal circulation (ECC). Measurements were obtained at baseline (BL), during VF at ECC 4 and 8 min, and post-resuscitation (PR). Each group had eight pigs at baseline. Numbers in brackets indicate when sample size decreased from the initial eight or preceding ones. Shown are the ratio between myocardial phosphocreatine and creatine (pCr/Cr), myocardial lactate content, and (in the inset) the relationship between lactate and pCr/Cr ratio at ECC 8 min. The regression line represents an exponential decay function (R^2^ = 0.63, *p* < 0.001). Mean ± SEM; * *p* < 0.05, ^‡^ *p* < 0.001 vs. control by Student’s *t*-test (adapted with permission from Ayoub et al. [6]).

As shown in Figure 9, myocardial tissue measurements indicated that administration of zoniporide prevented progressive loss of oxidative phosphorylation during the interval of simulated resuscitation. This effect was evidenced by a higher phosphocreatine-to-creatine (pCr/Cr) ratio, higher ATP/ADP ratio, and lesser increases in adenosine in animals treated with zoniporide. These metabolic benefits are consistent with the regeneration of ADP into ATP by mitochondria instead of downstream degradation to adenosine, with the newly formed ATP being used to regenerate creatine phosphate; all indicative of preserved mitochondrial bioenergetic function [6]. These changes were accompanied by amelioration of myocardial lactate increases, attaining levels which were inversely proportional to the pCr/Cr ratio at 8 min of VF and extracorporeal circulation (Figure 9), suggesting a shift away from anaerobic metabolism consequent to preservation of mitochondrial bioenergetic function in pigs treated with zoniporide.

These energy effects are consistent with NHE-1 inhibition protecting mitochondrial bioenergetic function—probably as a result of limiting mitochondrial Ca^2+^ overload—and supportive of the concept that preservation of left ventricular distensibility during resuscitation is likely to stem from lesser cytosolic and mitochondrial Ca^2+^ overload, correspondingly impacting cardiomyocyte relaxation and energy generation.

An additional consideration particularly relevant to cardiac resuscitation is the effect that changes in extracellular pH have on reperfusion injury after NHE-1 activation. We recently reported in our rat model if VF and closed-chest resuscitation a profound detrimental myocardial effect caused by the systemic administration of a buffer solution immediately after return of spontaneous circulation [27]. The buffer solution increased blood pH to ~7.70 units worsening post-resuscitation myocardial dysfunction and reducing survival. There was also a prominent increase in plasma cytochrome *c*, which is a mitochondrial protein responsible for the transfer of electrons from complex III to complex IV of the electron transport chain. Its release to the plasma is associated with the severity of myocardial injury after cardiac arrest [43,59]. Buffer administration was also associated with lactatemia attaining levels substantially higher than expected from cardiac arrest. We attributed these effects to intensification of the Na^+^-H+ exchange upon buffering of the extracellular pH with the adverse myocardial effect consequent to intensification of the Na^+^–driven cytosolic and mitochondrial Ca^2+^ overload. The excessive lactatemia is best explained by acceleration of the glycolytic pathway after buffer administration and attenuation of the intracellular acidosis given the pH-dependency of phosphofructokinase activity, which is the rate-limit enzyme of the glycolytic pathway [60,61,62]. Concomitant mitochondrial bioenergetic dysfunction precluding pyruvate utilization would contribute to the intensity of the observed lactatemia. All these effects were markedly attenuated when the NHE-1 inhibitor zoniporide was given (during CPR) before administration of the buffer solution. These observations are clinically relevant as the administration of sodium bicarbonate during pediatric cardiac resuscitation has been associated with worsened survival [63,64].

## 5. Clinical Translation of NHE-1 Inhibitors

Only a handful of studies—sponsored by pharmaceutical companies—have been conducted examining the effects of NHE-1 inhibition in humans. These studies, however, have examined conditions other than cardiac arrest including effects in patients undergoing coronary interventions for acute myocardial infarction [65,66,67] and during coronary artery bypass surgery (CABG) [66,68]. The studies in acute myocardial infarction were collectively inconclusive, with only one study showing myocardial benefits [65]. Of the studies in patients undergoing CABG, the EXPEDITION trial [68] demonstrated a prominent myocardial protective effect of cariporide reducing the incidence of post-operative myocardial infarction from 18.9% in the placebo group to 14.4% in the cariporide group with high statistical significance. Unfortunately, and unexpectedly, patients who received cariporide had a higher incidence of occlusive strokes. This adverse effect had not been reported in any of the other clinical trials or in animal models and is presumed to be unrelated to the mode of action but to its mode of administration—high dose and prolonged infusion [68,69]. The unexpected adverse effect observed in the EXPEDITION trial dampened the enthusiasm for further clinical development of NHE-1 inhibitors for cardiovascular conditions including cardiac resuscitation. Yet, the findings herein discussed, and the cardiac effect reported in the EXPEDITION trial, are highly supportive of potential clinically relevant effects of NHE-1 inhibitors.

## 6. Conclusions

We propose that the preclinical findings herein discussed are relevant to cardiac resuscitation in humans given the highly phylogenetically conserved nature of NHE-1, its physiological role, and its pathophysiological significance during ischemia and reperfusion. Functionally, preservation of left ventricular distensibility during CPR, greater electrical stability upon return of spontaneous circulation, attenuation of post-resuscitation myocardial dysfunction, and amelioration of the adverse effects or epinephrine are all highly desirable effects expected to improve outcome from cardiac arrest. Clinical studies determining the extent to which these effects can be translated to humans during cardiac resuscitation are eagerly awaited.
